# Physical mapping of repetitive oligonucleotides facilitates the establishment of a genome map-based karyotype to identify chromosomal variations in peanut

**DOI:** 10.1186/s12870-021-02875-0

**Published:** 2021-02-20

**Authors:** Liuyang Fu, Qian Wang, Lina Li, Tao Lang, Junjia Guo, Siyu Wang, Ziqi Sun, Suoyi Han, Bingyan Huang, Wenzhao Dong, Xinyou Zhang, Pei Du

**Affiliations:** 1 School of Life Sciences, Zhengzhou University, Zhengzhou, 450001 Henan China; 2 Henan Academy of Crop Molecular Breeding, Henan Academy of Agricultural Sciences/Key Laboratory of Oil Crops in Huang-Huai-Hai Plains, Ministry of Agriculture/Henan Provincial Key Laboratory for Oil Crops Improvement, Zhengzhou, 450002 Henan China; 3 Institute of Biotechnology and Nuclear Technology, Sichuan Academy of Agricultural Sciences, Chengdu, 610061 Sichuan China

**Keywords:** Peanut, TRs, Oligos, FISH, Karyotype, Chromosomal variants, Reference sequence

## Abstract

**Background:**

Chromosomal variants play important roles in crop breeding and genetic research. The development of single-stranded oligonucleotide (oligo) probes simplifies the process of fluorescence in situ hybridization (FISH) and facilitates chromosomal identification in many species. Genome sequencing provides rich resources for the development of oligo probes. However, little progress has been made in peanut due to the lack of efficient chromosomal markers. Until now, the identification of chromosomal variants in peanut has remained a challenge.

**Results:**

A total of 114 new oligo probes were developed based on the genome-wide tandem repeats (TRs) identified from the reference sequences of the peanut variety Tifrunner (AABB, 2*n =* 4x = 40) and the diploid species *Arachis ipaensis* (BB, 2*n =* 2x = 20). These oligo probes were classified into 28 types based on their positions and overlapping signals in chromosomes. For each type, a representative oligo was selected and modified with green fluorescein 6-carboxyfluorescein (FAM) or red fluorescein 6-carboxytetramethylrhodamine (TAMRA). Two cocktails, Multiplex #3 and Multiplex #4, were developed by pooling the fluorophore conjugated probes. Multiplex #3 included FAM-modified oligo TIF-439, oligo TIF-185-1, oligo TIF-134-3 and oligo TIF-165. Multiplex #4 included TAMRA-modified oligo Ipa-1162, oligo Ipa-1137, oligo DP-1 and oligo DP-5. Each cocktail enabled the establishment of a genome map-based karyotype after sequential FISH/genomic in situ hybridization (GISH) and in silico mapping. Furthermore, we identified 14 chromosomal variants of the peanut induced by radiation exposure. A total of 28 representative probes were further chromosomally mapped onto the new karyotype. Among the probes, eight were mapped in the secondary constrictions, intercalary and terminal regions; four were B genome-specific; one was chromosome-specific; and the remaining 15 were extensively mapped in the pericentric regions of the chromosomes.

**Conclusions:**

The development of new oligo probes provides an effective set of tools which can be used to distinguish the various chromosomes of the peanut. Physical mapping by FISH reveals the genomic organization of repetitive oligos in peanut chromosomes. A genome map-based karyotype was established and used for the identification of chromosome variations in peanut following comparisons with their reference sequence positions.

**Supplementary Information:**

The online version contains supplementary material available at 10.1186/s12870-021-02875-0.

## Background

Chromosomal variations, such as translocations, inversions, duplications and deletions play important roles in crop breeding and genetic research. Such variations are usually induced by physical [1–3], chemical [4, 5] and genetic factors [6]. A large number of chromosomal variants have been created and utilized in tomato [1], wheat [4], corn [7], cotton [8] and other crops.

Cultivated peanut (*Arachis hypogaea* L., 2*n =* 4x = 40, genome AABB) is grown worldwide as a major oilseed and cash crop. In 2018, the total production of peanut worldwide was 45,950,900 Metric tons, with an average yield of 1900.20 kg/ha [9]. Many peanut gene mutants have been created and investigated [10–13]. Candidate genes controlling pod width [14], seed coat color [15] and semi-dwarf status [16] have been identified by transcriptome sequencing of the gene mutants. However, only a few chromosome variations have been reported due to the lack of detection methods and reference genomes [17].

The traditional methods of cytological analysis of chromosomal variations include chromosome C-banding [17], genomic in situ hybridization (GISH) [18] and fluorescence in situ hybridization (FISH) using plasmid clones of TRs [19–21] or retransposons [22]. These methods have been used to establish karyotypes and to reveal both chromosomal structure and evolution. However, the karyotype resolution based on these methods is too low and insufficient to identify chromosomal variants in peanut. Moreover, the aforementioned methodologies are time-consuming and expensive, thereby limiting their wide application and efficiency in practice.

The recently developed single-stranded oligonucleotide FISH (SSON FISH) method provides a simple and efficient way to identify chromosomes in many species including wheat [23, 24], rye [25], barley [26], maize [27], rice [28], potato [29] and cucumber [30]. The development of genome-wide TR oligos has considerably improved the resolution of karyotypes, which has facilitated the accurate identification of chromosomal variants [23, 31, 32]. Additionally, physical mapping of TRs not only reveals genome evolution but can also guide sequence assembly in chromosomal regions with a high copy-number of repetitive sequences [23, 33].

In the peanut, Du et al. [34] applied SSON FISH using eight synthesized oligo probes to karyotype different peanut varieties and to identify a chromosome translocation line. A more efficient oligo dye staining method was further developed and applied to a large number of peanut samples [35]; however, limitations resulting from the small number of probes hindered karyotype resolution. Therefore, it is necessary to mine the peanut genome for TRs covering all the chromosomal regions to improve karyotype resolution.

Recently, the genome sequencing of the peanut [36–38] and its wild donor species, *A. duranensis* and *A. ipaensis* [39], have been completed. The comprehensive sequencing data enables the mining of the whole peanut genome for representative TRs covering all chromosomes. The homologous chromosomes are numbered based on genetic linkage maps in the reference genome map of the peanut [36]. However, the allocated chromosome numbers in the reference map do not correspond to the actual chromosomes in cytogenetic karyotypes [17, 18, 34]. Thus, a link between the genetic and cytogenetic maps is currently unavailable.

This study aims to: 1) Mine representative oligos at a whole-genomic level based on the reference sequences of the peanut variety Tifrunner (AABB) [36] and *A. ipaensis* (BB) [39]; 2) Establish a genome map-based karyotype of Tifrunner and physically map repetitive oligos to reveal the genomic organization in peanut; and 3) Use the karyotype to identify chromosomal variations induced by radiation exposure in peanut.

## Results

### Developing repetitive oligo probes from the whole genome sequencing data of peanut

Genome sequences of Tifrunner and *A. ipaensis* were analyzed using the Tandem Repeats Finder (TRF) [40], resulting in 4595 and 894 repetitive sequences, respectively. The lengths of these sequences varied between 4 bp and 723 bp while copy numbers varied between 50 and 29,162. After CD-HIT [41] elimination, a total of 80 and 35 TRs belonging to Tifrunner and *A. ipaensis*, respectively, were selected for further development of the oligos.

**Fig. 1:**
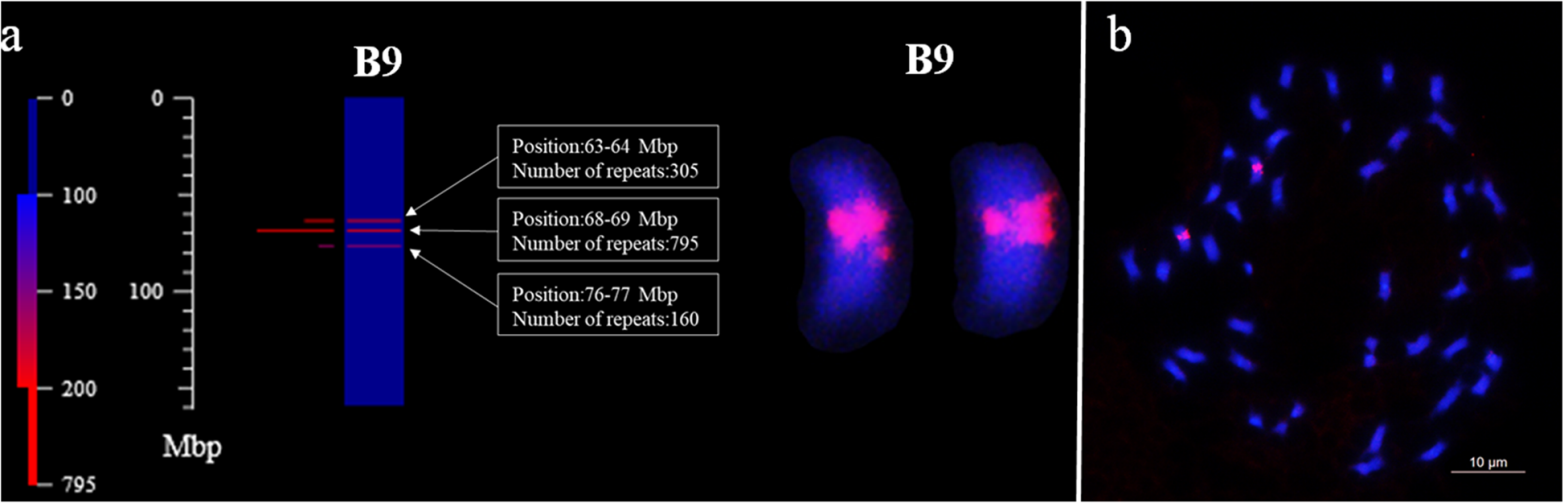
Confirmation of the orientation of chromosome B9 of cultivar Tifrunner. The chromosome plot is drawn using the Plot tool in the B2DSC server. **a** The red lines on the chromosome plot indicate that the consensus sequence of Ipa-1463 hits the sequences in the 63–77 Mbp region on the chromosome B9. **b** The location of the Ipa-1463 signals on the chromosome B9. Scale bar: 10 μm

**Fig. 2:**
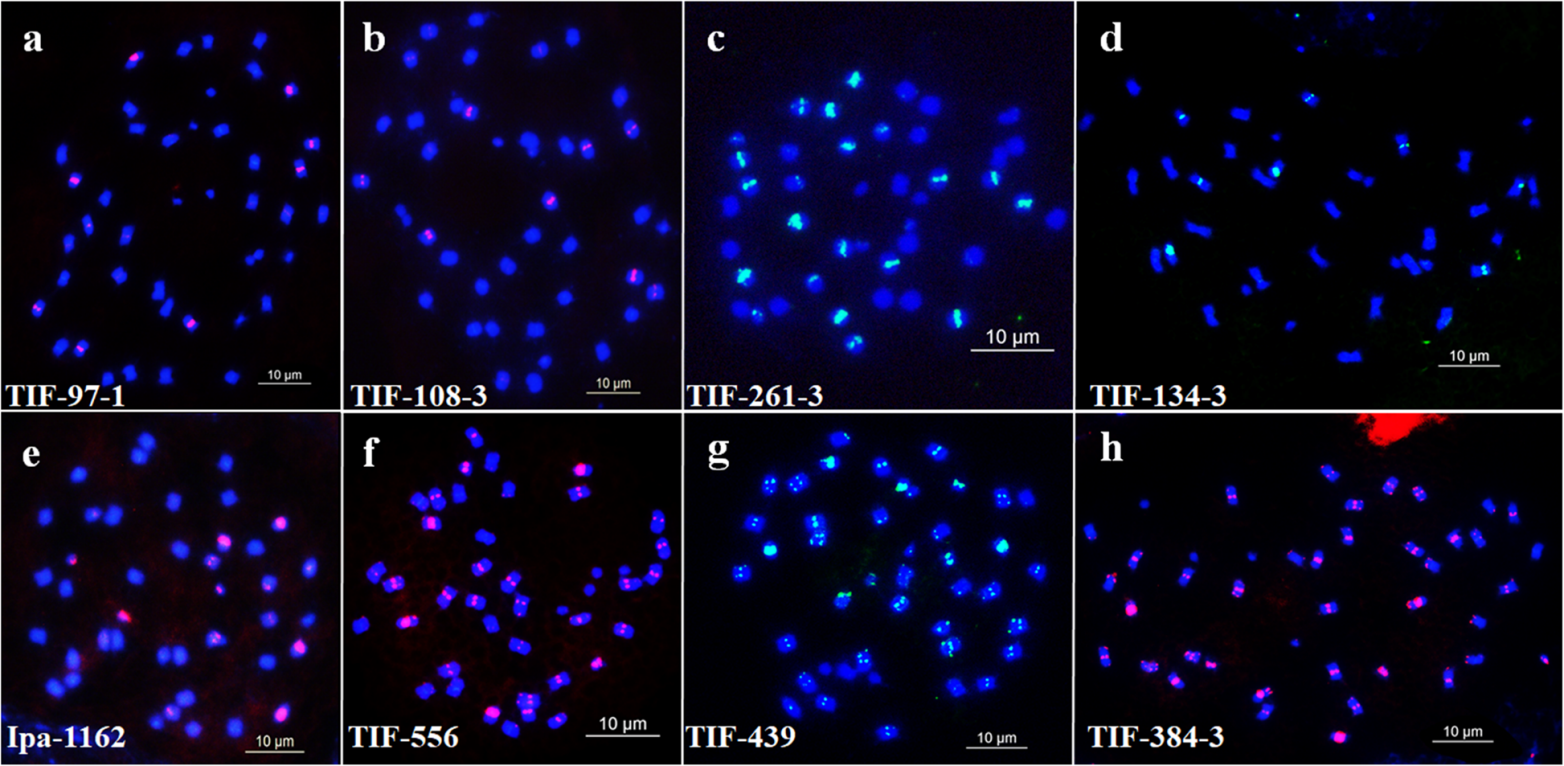
FISH of each probe on the chromosomes of the cultivar Tifrunner based on fluorescence in situ hybridization (**a**) TIF-97-1 (red, signals); **b** TIF-108-3 (red, signals); **c** TIF-261-3 (green, signals); **d** TIF-134-3 (green, signals); **e** Ipa-1162 (red, signals); **f** TIF-556 (red, signals); **g** TIF-439 (green, signals); **h** TIF-384-3 (red, signals). Scale bar: 10 μm

A total of 249 oligos were designed using Oligo 7 [42] and mapped in silico based upon the reference sequences of Tifrunner using B2DSC [31]. The designed oligos were first labeled via a random primer labeling method [27] and hybridized with the chromosomes of peanut. We observed by microscopy that 114 of the designed oligos produced clear signals in different positions of the chromosomes of Tifrunner (Fig. 1, Fig. 2, Table S1). The 114 oligo probes were categorized into 28 types based upon the pattern and position distribution observed by FISH (Table S2); oligos with the same position and overlapping signals in the karyotype were classified as the same type. For each of the 28 types, a single oligo was selected and further modified with 6-carboxyfluorescein (FAM) or 6-carboxytetramethylrhodamine (TAMRA). Figure 1 shows the results of oligo Ipa-1463 after in silico mapping and FISH. In chromosome plots, 1260 copies were observed in the 63–77 Mbp region of chromosome B9 (Fig. 1a). The FISH analysis confirmed that the signals of oligo Ipa-1463 were only present in one pair of chromosomes at a similar region. Thus, we concluded that this chromosome corresponded with chromosome B9 in the reference sequence (Fig. 1b).

### Development of a genome map-based karyotype of Tifrunner

**Fig. 3:**
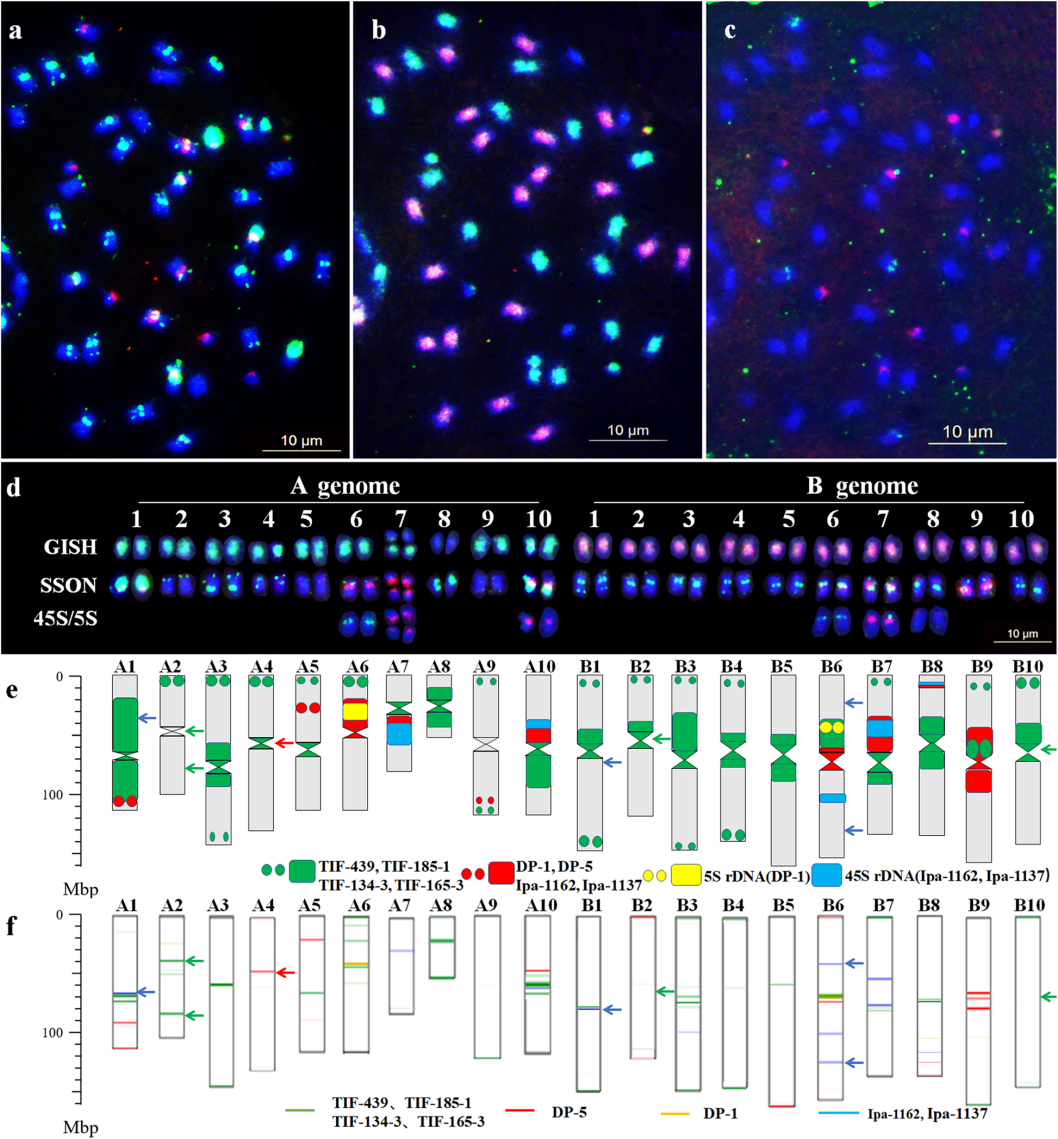
Sequential fluorescence in situ hybridization/genomic in situ hybridization (FISH/GISH) karyotype and chromosome plots of the cultivar Tifrunner. **a** FISH using Multiplex #3 (green) and Multiplex #4 (red); **b** GISH using genomic DNA of *A. duranensis* (green) and *A. ipaensis* (red); **c** FISH using 45S rDNA (red) and 5S rDNA (green) as the probes; **d** Karyotypes corresponding to chromosomes in the sequencing map of Tifrunner; SSON indicates the FISH karyotype using Multiplex #3 (green) and Multiplex #4 (red); GISH indicates the FISH karyotype using total genomic DNAs of *A. duranensis* (green) and *A. ipaensis* (red); 45S/5S indicates the FISH karyotype using 45S rDNA (red) and 5S rDNA (green); **e** Idiogram karyotype of Tifrunner; **f** chromosome plots of Tifrunner; blue arrows in (**e**) and (**f**) indicate the non-correspondent signals of oligos Ipa-1162 and Ipa-1137 between the karyotype and chromosome plots; green arrows in (**e**) and (**f**) indicate the non-correspondent signals of oligos TIF-439, TIF-185-1, TIF-134-3 and TIF-165-3 between the karyotype and chromosome plots; and red arrows in (**e**) and (**f**) indicate the non-correspondent signals of oligos DP-5 between the karyotype and chromosome plots. Scale bar: 10 μm

Based on the unique patterns and sequence composition of the 28 oligo probes, two new oligo probe cocktails, Multiplex #3 and Multiplex #4, were developed using eight synthesized oligos. Multiplex #3 included FAM-modified TIF-439, TIF-185-1, TIF-134-3 and TIF-165-3. Multiplex #4 included TAMRA-modified Ipa-1162, Ipa-1137, DP-1 and DP-5. Both DP-1 and DP-5 were derived from a previous study by Du et al. [34]. Following the sequential FISH/GISH with Multiplex #3 and Multiplex #4, total genomic DNAs of *A. duranensis* and *A. ipaensis*, 45S and 5S rDNAs as the probes, a robust karyotype of Tifrunner was established (Fig. 3a–e).

Comparing the distributions of the eight oligos in the karyotype and chromosome plots, we found that most signal sites and intensities in the actual chromosomes corresponded well with their respective positions and copy numbers in the reference sequence (Fig. S1). Additionally, a genome map-based karyotype of Tifrunner was established. The actual chromosomes in the karyotype were renumbered as A1 ~ A10 and B1 ~ B10, according to their pseudo-molecule numbers in the genome map of Tifrunner [36]. We found short arms of some chromosomes in the karyotype, such as A1 and B7, were not positioned on the short arm (upper arm) in the genome map. In order to facilitate understanding, the long arm (L) and short arm (S) in this study were assigned according to the upper arm and lower arm of the genome map. Based upon this karyotype, nine significant non-correspondent signals were observed across seven chromosomes (Fig. 3e–f). For example, the oligos Ipa-1162 and Ipa-1137 evidently had distribution sites on chromosomes A1 and B1 in the chromosome plots, but no signal was observed upon probe hybridization to the actual chromosomes. In contrast, oligos TIF-439, TIF-185-1, TIF-134-3 and TIF-165-3 produced strong signals in the centromeric regions of chromosomes B2 and B10, but were not in silico mapped in the chromosome plots (Fig. 3d–f, Fig. S1).

To validate the genome map-based karyotype, two chromosome-specific single-copy sequence oligo libraries, L1A-1 and L3A-1, from the short arm of chromosomes A1 and A3 in the genome map, were used for sequential FISH/GISH analysis. The libraries were combined with the Multiplex #3, Multiplex #4 and total genomic DNAs of *A. duranensis* and *A. ipaensis* as the probes and used for the subsequent analysis (Fig. S2). The specific signals of the two chromosome-specific oligo libraries were clearly observed on the two expected chromosomes, indicating a considerable correspondence between the actual chromosomes of the karyotype and the genome maps.

Among the A subgenome chromosomes of this karyotype, chromosomes A1 and A8 both contained intense green signals in the centromeric regions, while A1 also had strong red signals in the terminal region of its long arm. Chromosomes A6, A7 and A10 had 45S or 5S rDNAs sites. Chromosomes A2, A3 and A4 had green signals in the terminal regions of the short arms, while A3 and A4 had green signals in the centromeric regions. Chromosome A5 had red signals within the short arm, while A9 had red signals at the subtelomeric region of the long arm. Among the B subgenome, chromosomes B6, B7 and B8 showed signals of the probes for 45S rDNA or 5S rDNA. Chromosome B9 and B3 had red and green signals in the centromeric regions, respectively. The other chromosomes had green signals either in the centromeric or telomeric regions with varying intensities (Fig. 3e). Based on the unique patterns observed, all chromosomes could be clearly differentiated in the karyotype.

### Chromosome allocation of the oligo probe

**Fig. 4:**
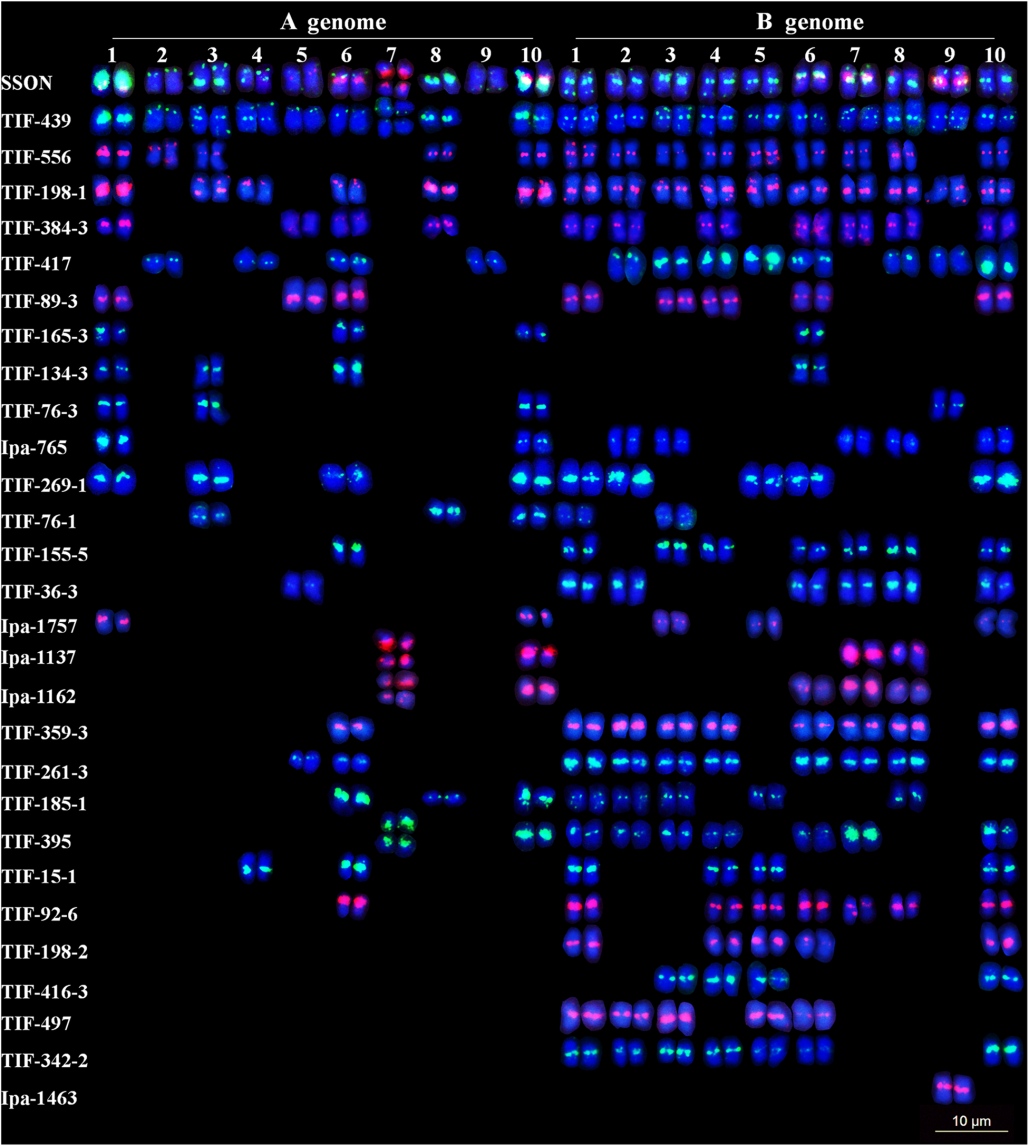
Karyotypes of all 28 oligos representing the 28 distribution patterns by sequential fluorescence in situ hybridization (FISH) using 45S rDNA (red), 5S rDNA (green), *A. duranensis* (green) and *A. ipaensis* (red) total genomic DNAs as probes of the cultivar Tifrunner. Scale bar: 10 μm

To map TRs in the genome map-based karyotype of Tifrunner, the 28 representative oligos were analyzed by both FISH and in silico mapping (Fig. 4). The 28 oligos produced more signals on the B subgenome chromosomes than on those of the A subgenome (Fig. 4). Among the 28 oligos, six (TIF-165-3, TIF-439, TIF-556, TIF-198-1, TIF-384-3 and TIF-185-1) produced signals in the interstitial or terminal regions of the chromosomes. Four oligos (TIF-198-2, TIF-416-3, TIF-497 and TIF-342-2) had signals exclusively on the B subgenome, indicating that these oligos are specific to the B subgenome. Two oligos (Ipa-1137 and Ipa-1162) had signals only at the secondary constrictions, which fully overlapped with the signals of 45S rDNA, following sequential FISH. Oligo Ipa-1463 exclusively showed signals on one pair of chromosomes, which indicates that it is chromosome-specific. The other 15 oligos had signals located within the pericentric regions (Fig. S3).

Among the 28 oligos physically mapped via FISH, the distributions of 22 oligos were the same or similar to those in the in silico mapping results (Fig. 4, Fig. S4). However, six oligos (TIF-89-3, TIF-155-5, TIF-198-1, TIF-359-3, TIF-76-1 and Ipa-1757) showed significant differences between the two maps. For example, TIF-89-3 and TIF-155-5 were in silico mapped to two pairs of chromosomes with a high number of copies. However, eight pairs of chromosomes evidently showed FISH signals. Similarly, TIF-198-1 was in silico mapped to only one pair of chromosomes, but produced signals on 16 pairs of chromosomes. In contrast, obvious sites of TIF-76-1 were in silico mapped to 13 pairs of chromosomes but produced FISH signals on five pairs of chromosomes. This may indicate that TRs were unambiguously not assembled within the genome map of Tifrunner.

### Identification of chromosomal variations in Silihong (SLH) induced by radiation exposure

**Fig. 5:**
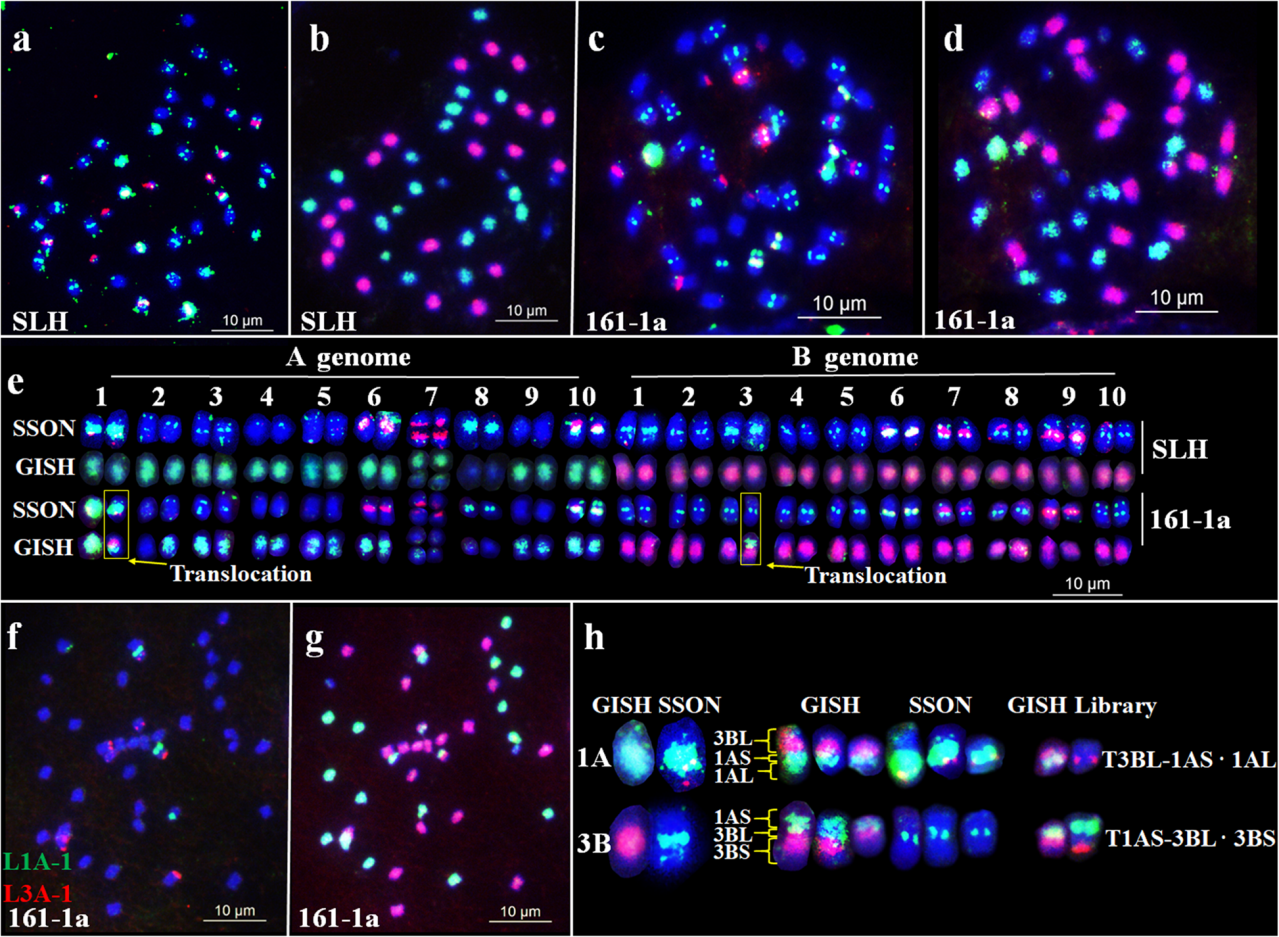
Chromosome variants detected in the radiation-induced M_1_ plant 161-1a of the peanut cultivar Silihong (SLH) after sequential fluorescence in situ hybridization/genomic in situ hybridization (FISH/GISH). **a**–**d** Results of sequential FISH/GISH in SLH (**a**–**b**) and M_1_ plant 161-1a (**c**–**d**), using Multiplex #3 (green) and Multiplex #4 (red) (a and c); *A. duranensis* genomic DNA (b and d; green); *A. ipaensis* genomic DNA (b and d; red). **e** Karyotypes of SLH and 161-1a. **f**–**g** Sequential FISH/GISH using probe Libraries L1A-1 (green) and L3A-1 (red). **h** Translocated chromosomes in 161-1a. SSON indicates the FISH karyotype using Multiplex #3 (green) and Multiplex #4 (red); GISH indicates the FISH karyotype using total genomic DNAs of *A. duranensis* (green) and *A. ipaensis* (red). Scale bar: 10 μm

To check chromosomal variations in peanut, sequential FISH and GISH were conducted in Chinese variety SLH and 70 radiation-induced M_1_ SLH plants with Multiplex #3 and Multiplex #4, total genomic DNAs of *A. duranensis* and *A. ipaensis*, 45S and 5S rDNAs as the probes (Fig. 5). The fourteen M_1_ plants used within this study showed chromosomal variations. For example, in plant 161-1a one reciprocal translocation was evident, which likely occurred between chromosomes 1A and 3B. The segment of one chromosome translocation from the A subgenome had extensive green signals spanning the entire arm and red signals at the terminal end of the chromosome. This pattern was similar to that of the signals on the centromere and the long arm (L) of 1A. The signal distribution pattern of another segment was like the pattern of 3B from the end of short arm (S) to centromere, which indicates that they were T 3BL-1AS·1AL and T 1AS·3BL-3BS. Additional FISH was performed using the single-copy oligo library probes L1A-1 and L3A-1 which exclusively hybridized to the upper arms of 1A and 3A. The results of this experiment confirmed the identity of the two translocated chromosomes (Fig. 5f–h).

**Fig. 6:**
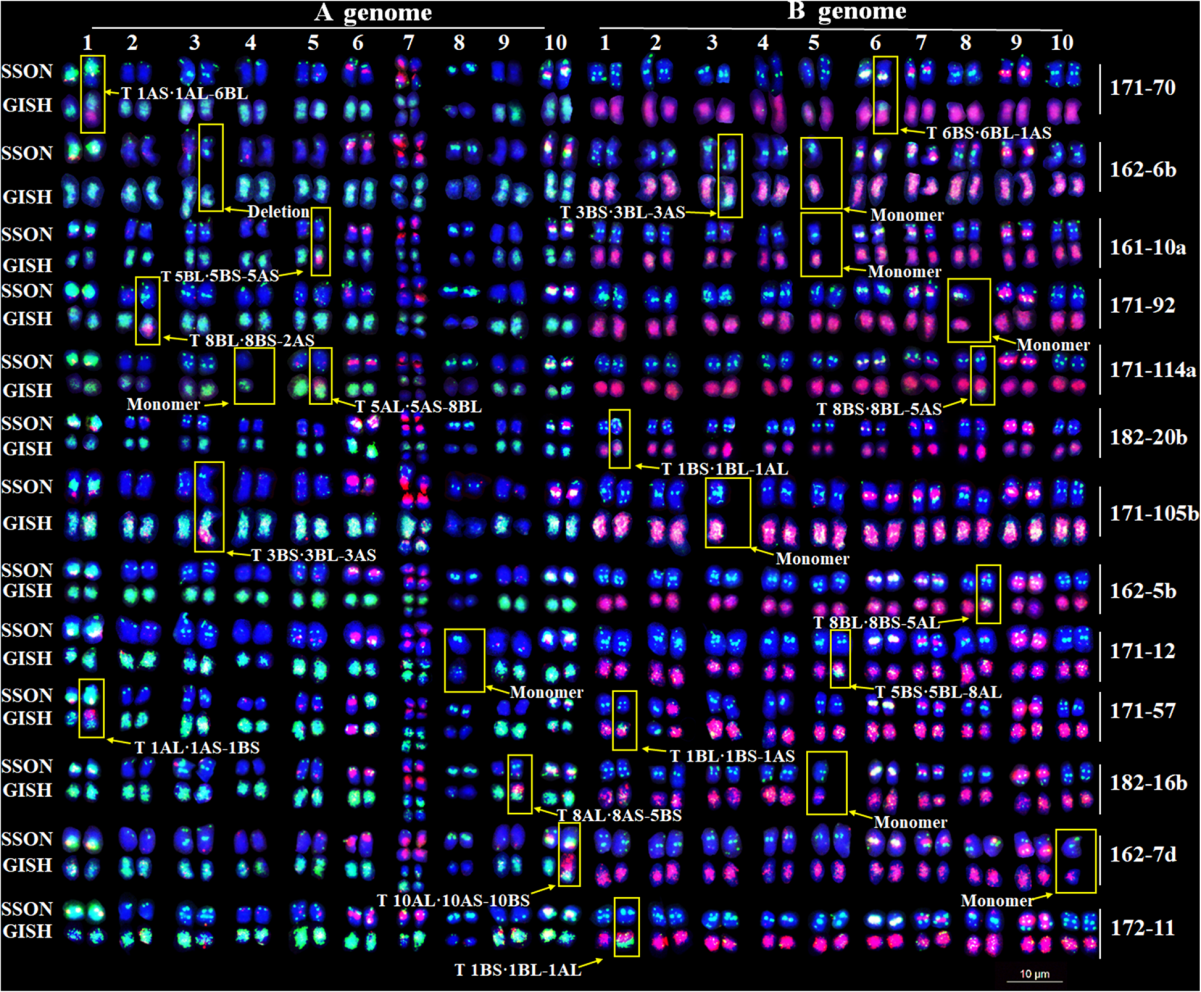
Karyotypes and translocated chromosomes of radiation-induced M_1_ plants. SSONs show the FISH karyotype using SSON probes Multiplex #3 (green) and Multiplex #4 (red); GISH show the FISH karyotype using total genomic DNAs of *A. duranensis* (green) and *A. ipaensis* (red). Scale bar: 10 μm

A total of 13 other plants were identified to have 17 translocations, one deletion and eight monosomic chromosomes (Fig. S5). Among the identified chromosomal variations, eight translocations were observed between homoeologous chromosomes and nine translocations were observed between non-homologous chromosomes. Chromosomes 1, 3 and 5 showed a greater number of translocations (Fig. 6).

## Discussion

### Applications of the new oligo probes

The TRs comprise a large proportion of the plant genome. In the past, DNA repeats were regarded as genomic ‘junk’ [43]. However, in recent years various studies have shown that TRs could be used to gain a better understanding of evolutionary history and genomic composition [44]. TRs also serve as placeholders for epigenetic signals that govern heterochromatin formation, and may have function in repairing double-strand DNA breaks [44, 45].

Many repetitive DNA elements generate specific distribution patterns on chromosomes among various species. These elements are the most common sources of probes for chromosome identification and cytogenetic studies in plants [23, 46, 47]. Du et al. [34] developed eight oligo probes of peanut based on genome skimming of *A. duranensis*, established a high-resolution karyotype, and revealed genome relationships among eight *Arachis* species. However, the oligo probes used in their study were neither genome-specific nor chromosome-specific. Furthermore, they did not cover the interstitial regions of peanut chromosomes.

In the present study, 114 new oligos covering different regions of peanut chromosomes were developed. Twenty-eight oligos, representing 28 chromosome distribution patterns, were selected and mapped onto peanut chromosomes. Eight oligos could be located in the secondary constrictions, interstitial, or terminal regions; four were B genome-specific and one was chromosome-specific. These findings indicate that these probes might be useful for the precise identification of peanut chromosome translocations with considerably high resolution provided that various combinations of oligo probes were used. In comparison with previously reported markers, such as retrotransposons [48], 45S and 5S rDNAs plasmid clones [49] and a few bacterial artificial chromosome (BAC) clones [21], the 28 newly identified oligos produced signals covering different regions of chromosomes. Additionally, the development of the Multiplex #3 and Multiplex #4 assays considerably simplified the FISH procedure. These new developments would facilitate chromosome engineering of peanut and support the development of further applications in the future.

Since repetitive sequences are considered to have evolved under different evolutionary pressures, comparative mapping of the repetitive sequences via FISH could facilitate phylogenetic analysis [33]. Based on repetitive sequences, the genome constitution of peanut as a tetraploid species has been supposed [21] and relationships among the A, B, K, F, E and H genomes of 51 *Arachis* species were clarified [35]. In addition, the 28 oligos identified in the present study would provide effective tools for genome evolutionary research of peanut and its relatives.

### Genome map-based karyotype of Tifrunner revealed specific characteristics in the distribution of repetitive sequences in peanut

Repetitive sequences in Tifrunner account for 74% of the assembled genome [36]. The recent sequencing of the peanut genomes [36–39] and the development in peanut chromosome research [19–22] have provided valuable information. However, the link between the reference genome map and the physical chromosome karyotype is still missing. Using the reference sequences of Tifrunner, oligos were in silico mapped and compared with the resulting FISH karyotype. Most of the repeat sequence positions and copy numbers in the reference map were the same or similar to their FISH signal distributions and intensities in the karyotype. As the result, a genome map-based karyotype of Tifrunner was established, in which all chromosomes were renumbered as indicated in the reference genome map.

Furthermore, our findings revealed that several repeats were inconsistently mapped with respect to the actual chromosomes and the in silico sequence. For example, TIF-89-3, TIF-155-5 and TIF-198-1 produced FISH signals in more chromosomes than expected, while TIF-76-1 was in silico mapped to more chromosome positions than those which were visualized by our FISH experiments. These results indicate that the assembly of the reference sequences of Tifrunner still may need additional refinement, particularly in the regions with a high copy number of TRs. The ambiguity in the assembly of high-copy elements is problematic in many species with large genomes. The present work highlights the need of further sequencing and assembly of the peanut genome.

### Importance of the identification of chromosomal variants for peanut research and breeding

Development and physical mapping of our newly developed 28 oligos have considerably improved the karyotype resolution of peanut, thereby facilitating a more efficient identification of chromosomal variations. In the present study, 14 out of 70 radiation-induced M_1_ plants were identified to have translocations, deletions and monosomic chromosomes. This indicates the potential of both the oligos identified and induction of chromosomal mutations through irradiation in peanut chromosome engineering. The chromosomal variations could be used for translocation or deletion mapping, radiation hybrid mapping and gene mapping of peanut as has been reported previously [50, 51].

Furthermore, translocations occurring between partially homologous chromosomes or heterologous chromosomes promote the exchange of DNA beyond homologous chromosomes in conventional breeding, which could lead to new beneficial traits. In future studies, we intend to identify additional translocations and produce inbred translocation lines to create homozygous translocations with beneficial genes. This research could facilitate the genetic improvement of peanut.

## Conclusions

Based on the reference sequences of Tifrunner and *A. ipaensis*, 114 new repetitive sequence oligos were developed and chromosomally positioned. They were classified into 28 types, mainly based on their positions and overlapping signals along the chromosomes. A total of 28 individual oligos, representing each of the 28 types, were selected and conjugated with FAM and TAMRA. Following distribution comparison of eight oligos on chromosome plots in the reference sequences of Tifrunner, a genome map-based FISH karyotype was constructed in which all chromosomes were renumbered as the references. The present karyotype has considerably improved resolution and can facilitate the identification of the 14 chromosomal variations of SLH. However, several TRs produced signals that were inconsistent with their copy numbers and positions in the reference sequences of Tifrunner. This indicates that the assembly of the reference sequences of Tifrunner still needs further refinement. The unique distribution patterns of the 28 oligos in the karyotype provide visible evidences for correction of such minor errors. Therefore, the present novel probes and karyotype provide effective tools for chromosome engineering and evolutionary studies of peanut.

## Methods

### Plant materials

Peanut (*A. hypogaea* L., 2*n =* 4x = 40) varieties, Tifrunner and SLH, were kept by Henan Academy of Crop Molecular Breeding, Henan Academy of Agricultural Sciences, China. To develop chromosomal variations, SLH plants at the flowering stage were irradiated with a dosage of 16 Gy of gamma ray using a ^60^Co source (Isotope Institute Co. Ltd., Henan Academy of Sciences). A total of 70 M_1_ plants were used for this study.

### Chromosome preparation

Chromosomes were prepared, as described by Du et al. [52] with minor adjustments. When the roots of the peanut plant reached about 2 cm long, root tips were cut and placed in 8-hydroxyquinoline for 3 h at 25 °C. They were subsequently treated with nitrous oxide (N_2_O) at a pressure of 0.8–1.2 MPa for 1.5 h and fixed in absolute ethanol-glacial acetic acid (3:1) solution for 12 h at 25 °C. The treated root tips were then stored in a refrigerator at − 20 °C until further use. The apex was resected and disintegrated in 45% glacial acetic acid for 2–3 min, compressed, and then frozen at − 80 °C in an ultra-low temperature refrigerator for 24 h. The root material was dehydrated in absolute ethanol for 12 h and air-dried for FISH.

### Design of oligo probes

The whole genome assembly sequences of Tifrunner and *A. ipaensis* were downloaded from PeanutBase (https://peanutbase.org/home). The sequences of both Tifrunner and *A. ipaensis* genomes were analyzed to obtain tandem repeat sequences using the Tandem Repeats Finder (TRF, version 4.09) [40] based on the methods of Tang et al. [31]. The following parameters were applied: match = 2; mismatch = 7; indel = 7; probability of match = 80; probability of indel = 10; min score = 50; and max period = 2000. Data with a period size > 4 and DNA copy number > 50 were obtained using a Python script. Tandem repeat sequences with > 75% identity were determined using the CD-HIT [41] for clustering and maintaining consensus for each cluster. Oligos with the lengths of 40–45 nt were then designed based on the tandem repeat sequences and Oligo 7 [42].

To determine the effectiveness of each designed probe, oligos were first physically mapped onto reference sequences of the chromosomes. The sequence of each oligo probe was aligned using the Nucleotide Basic Local Alignment Search Tool (blastn) in the B2DSC2 server against the Tifrunner genome. The blast results were filtered using pident = 90% and qcovhsp = 90%. The physical location information was drawn using the Plot tool in B2DSC. Oligos with a rich distribution of sites were synthesized by the General Biosystems Company (Anhui, China).

### FISH and sequential FISH analysis

Oligo probes were first labeled with biotin-16-dUTP or digoxigenin-11-dUTP using end labeling techniques. The 10-μl system included: 2 μl 5 × terminal deoxynucleotidyl transferase (TDT) buffer; 0.5 μl dATP; 3.5 μl dd H_2_O; 2 μl CoCl_2_; 0.5 μl TDT enzyme; 0.5 μl biotin-16-dUTP or digoxigenin-11-dUTP; and oligo (1 ng/μl) 1 μl; maintained at 37 °C for 15 min. After screening, 28 oligo probes were obtained (Table S2). All repetitive oligos were modified at the 5′-ends with TAMRA or FAM by General Biosystems Company (Anhui, China) for further use in FISH. To establish the karyotype, two multiplex probe cocktails, named Multiplex #3 and Multiplex #4, were developed. Multiplex #3 included FAM-modified TIF-439, TIF-185-1, TIF-134-3 and TIF-165-3; and Multiplex #4 included TAMRA-modified Ipa-1162, Ipa-1137, DP-1 and DP-5.

Two single-copy oligo libraries (L1A-1 and L3A-1) from chromosomes A1 and A3 were developed using genomic sequences of *A. duranensis* in PeanutBase according to the methods described by Du et al. [34]. Each single-copy oligo library was derived from the distal region of each chromosome. Libraries were synthesized by MYcroarray (Ann Arbor, MI, USA). The resulting libraries were amplified and labeled with biotin-16-dUTP or digoxigenin-11-dUTP according to the MYcroarry_MYtags labeling protocol.

The FISH and sequential FISH procedures followed those described by Du et al. [34]. Briefly, the slides were denatured in 70% formamide at 75 °C for 70 s. The hybridization solution, including 3 μl of each probe, was denatured for 13 min. The slides were immersed in the hybridization solution at 37 °C in a wet box for at least 12 h, and then washed 10 times with 2 × saline-sodium citrate at 42 °C. Slides were then stained with 4′, 6-diamidino-2-phenylindole (DAPI) and mounted with Mounting Medium.

Sequential FISH was performed to map the signals of oligo probes and correlate a sequenced chromosome with a cytologically identified chromosome. Thereafter, FISH was conducted using repetitive or single-copy oligo probes which were then washed to remove all signals before being dried. The GISH procedure was performed using total genomic DNAs of *A. duranensis* and *A. ipaensis* as the probes, and FISH was conducted using 45S and 5S rDNAs as the probes.

### Capture of images and analysis

The FISH images were captured using a Leica DM6000 fluorescence microscope (Leica) with a cooled charge-coupled device camera (Leica). Images were optimized for contrast and brightness using Adobe Photoshop. Approximately 3–5 cells of each accession were observed for karyotyping and chromosome diversity analysis. Most karyotypes were developed from single cells unless chromosomes showed overlapping.

## Supplementary Information

**Additional file 1: Fig. S1.** Physical mapping of oligo TIF-165-3 (a), DP-1(b), TIF-439(c), TIF-185-1(d), TIF-134-3(e), DP-5(f), Ipa-1162(g) and Ipa-1137(h) using the Plot tool in the B2DSC server. The green, red and blue lines on the chromosome plots indicate copy numbers of the oligo sequences; the length of the lines correspond to the number of copies.

**Additional file 2: Fig. S2.** Sequential fluorescence in situ hybridization/genomic in situ hybridization (FISH/GISH) using chromosome-specific oligo libraries L1A-1 (a, red) and L3A-1 (d, red); SSON probes Multiplex #3 (green) and Multiplex #4 (red) (b and e); and total genomic DNAs of *A. duranensis* (green) and *A. ipaensis* (red) (c and f) in the cultivar Tifrunner. Green and red arrows in (a) show chromosome A1 and B1 respectively; green and red arrows in (d) show chromosome A3 and B3 respectively. Scale bar: 10 μm.

**Additional file 3: Fig. S3.** Sequential FISH using oligo TAMRA-Ipa-1162 and TAMRA-Ipa-1137 (a, red) and the plasmid clone 45S rDNA (b, red) as probes. Scale bar: 10 μm.

**Additional file 4: Fig. S4.** Physical mapping of oligo TIF-89-3 (a), TIF-155-5 (b), TIF-198-1 (c), TIF-359-3 (d), TIF-76-1 (e) and Ipa-1757 (f) using the Plot tool in the B2DSC server. The length of the red lines on the chromosome plots indicate the copy numbers of the oligo sequences.

**Additional file 5: Fig. S5.** Chromosome aberration variants detected in the radiation-induced M_1_ plant 161-1a of peanut cultivar Silihong (SLH) after sequential FISH/GISH. FISH using Multiplex #3 (a, c, e, g, i, k, m, o, q, s, u, w and y); GISH using *A. duranensis* genomic DNA (green) and *A. ipaensis* genomic DNA (red) (b, d, f, h, j, h, j, l, n, p, r, t, v, x and z). Scale bar: 10 μm.

**Additional file 6: Table S1.** Details of the new oligo probes from the cultivar Tifrunner and *A. ipaensis*.

**Additional file 7: Table S2.** Oligo probes classification and chromosomal information.

## Data Availability

All the data pertaining to the present study have been included in table and/or figure form in the manuscript and authors are pleased to share analyzed/raw data and plant materials upon reasonable request. Other datasets supporting the conclusions of this article are included within the article and its additional files.

## Abbreviations

oligo: Oligonucleotide
FISH: Fluorescence in situ hybridization
TRs: Tandem repeats
FAM: 6-carboxyfluorescein
TAMRA: 6-carboxytetramethylrhodamine
GISH: Genomic in situ hybridization
SSON: Single-stranded oligonucleotide
TRF: Tandem Repeats Finder
SLH: Silihong
BAC: Bacterial artificial chromosomes
N_2_O: Nitrous oxide
TDT: Terminal deoxynucleotidyl transferase
DAPI: 4′, 6-diamidino-2-phenylindole
