# Supplementary figures and images for: Physical mapping of repetitive oligonucleotides facilitates the establishment of a genome map-based karyotype to identify chromosomal variations in peanut
Source: BMC Plant Biol. 2021 Feb 20;21:107. doi: 10.1186/s12870-021-02875-0 (PMC7896385; doi:10.1186/s12870-021-02875-0)

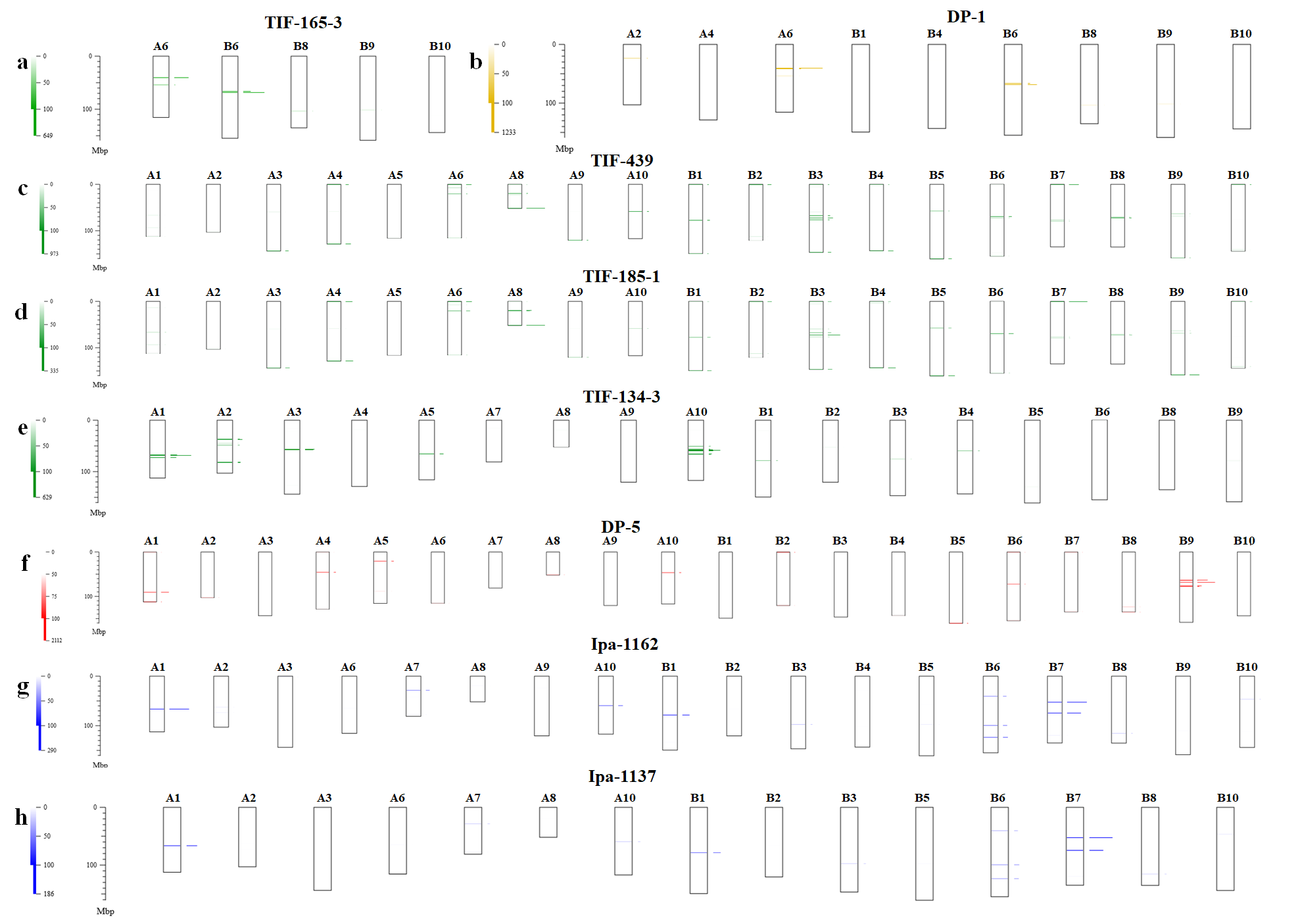

Supplement: Supplementary file 1 — Additional file 1: Fig. S1. Physical mapping of oligo TIF-165-3 (a), DP-1(b), TIF-439(c), TIF-185-1(d), TIF-134-3(e), DP-5(f), Ipa-1162(g) and Ipa-1137(h) using the Plot tool in the B2DSC server. The green, red and blue lines on the chromosome plots indicate copy numbers of the oligo sequences; the length of the lines correspond to the number of copies. [file 12870_2021_2875_MOESM1_ESM.tif]

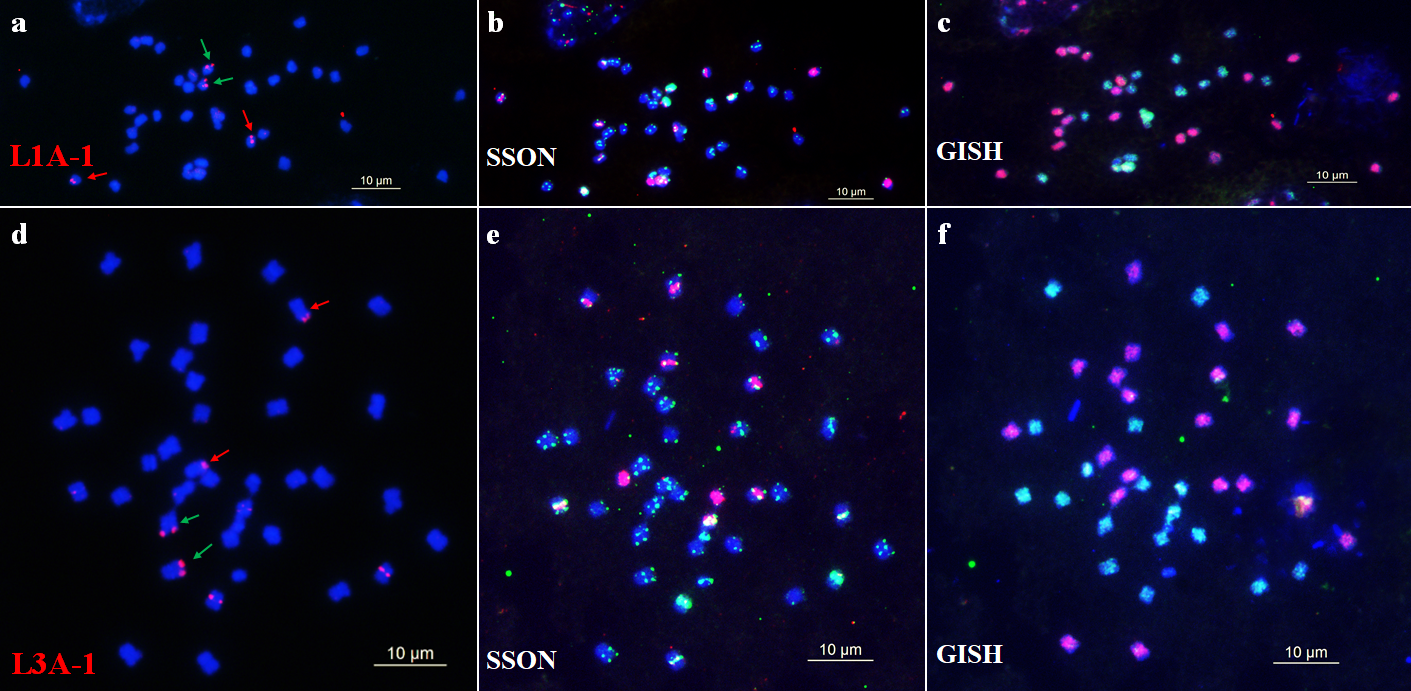

Supplement: Supplementary file 2 — Additional file 2: Fig. S2. Sequential fluorescence in situ hybridization/genomic in situ hybridization (FISH/GISH) using chromosome-specific oligo libraries L1A-1 (a, red) and L3A-1 (d, red); SSON probes Multiplex #3 (green) and Multiplex #4 (red) (b and e); and total genomic DNAs of A. duranensis (green) and A. ipaensis (red) (c and f) in the cultivar Tifrunner. Green and red arrows in (a) show chromosome A1 and B1 respectively; green and red arrows in (d) show chromosome A3 and B3 respectively. Scale bar: 10 μm. [file 12870_2021_2875_MOESM2_ESM.tif]

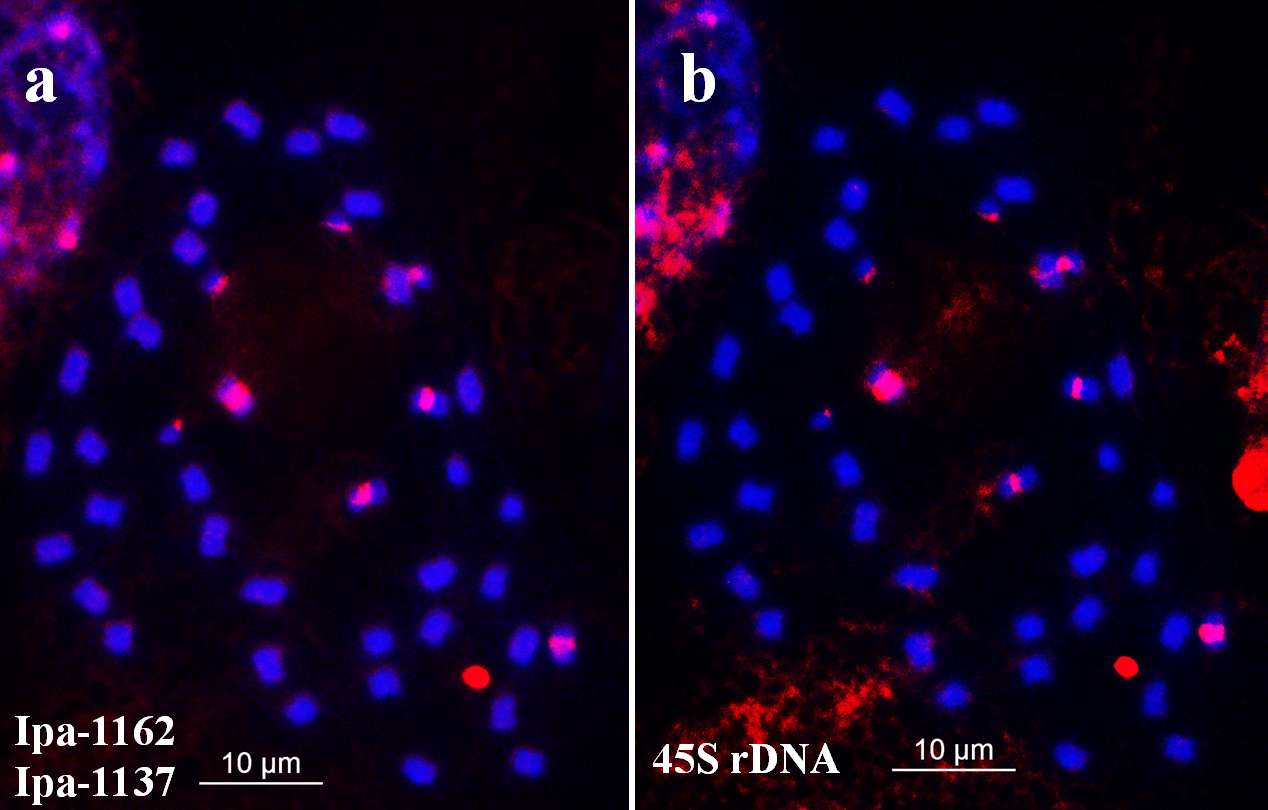

Supplement: Supplementary file 3 — Additional file 3: Fig. S3. Sequential FISH using oligo TAMRA-Ipa-1162 and TAMRA-Ipa-1137 (a, red) and the plasmid clone 45S rDNA (b, red) as probes. Scale bar: 10 μm. [file 12870_2021_2875_MOESM3_ESM.tif]

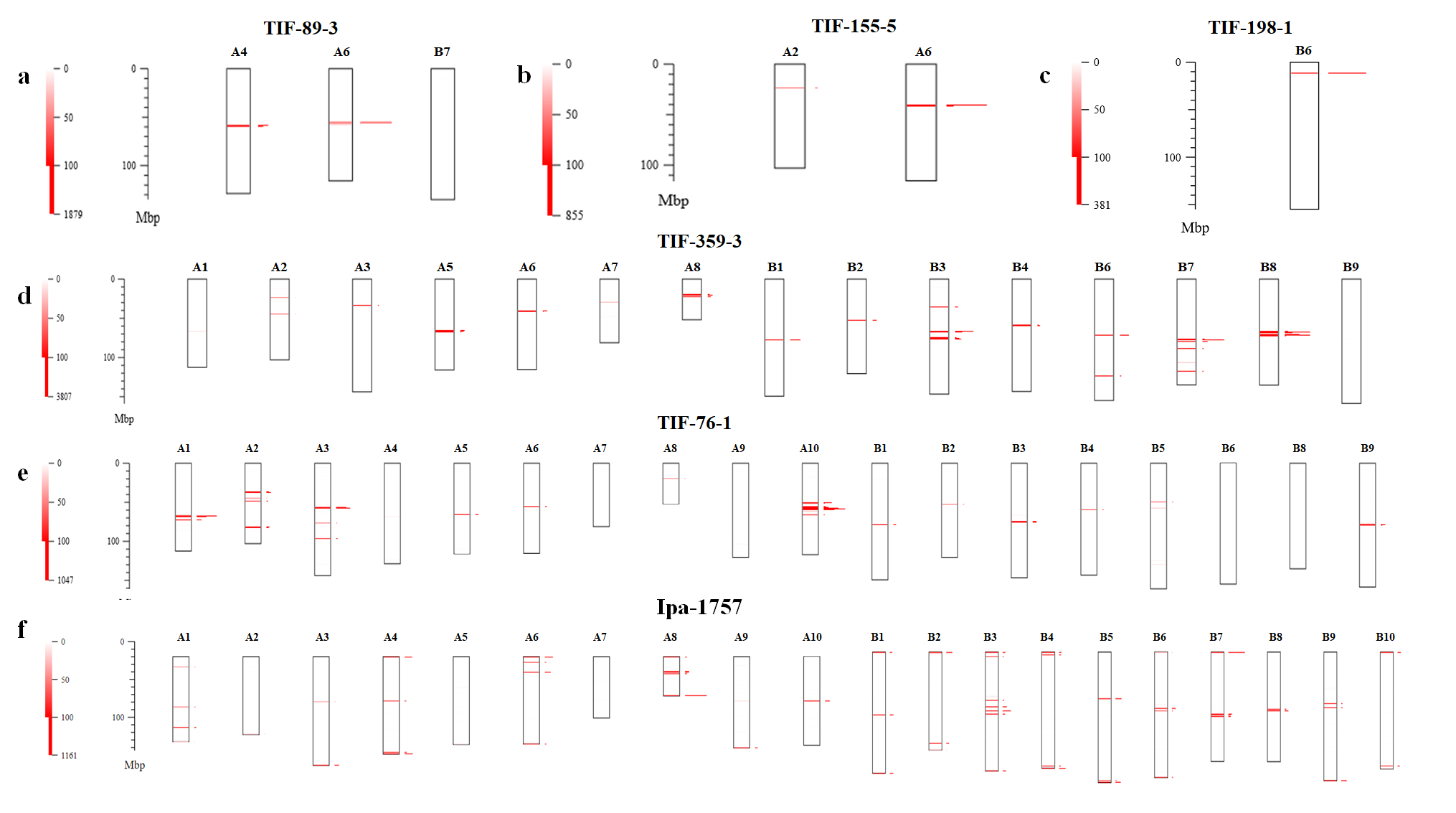

Supplement: Supplementary file 4 — Additional file 4: Fig. S4. Physical mapping of oligo TIF-89-3 (a), TIF-155-5 (b), TIF-198-1 (c), TIF-359-3 (d), TIF-76-1 (e) and Ipa-1757 (f) using the Plot tool in the B2DSC server. The length of the red lines on the chromosome plots indicate the copy numbers of the oligo sequences. [file 12870_2021_2875_MOESM4_ESM.tif]

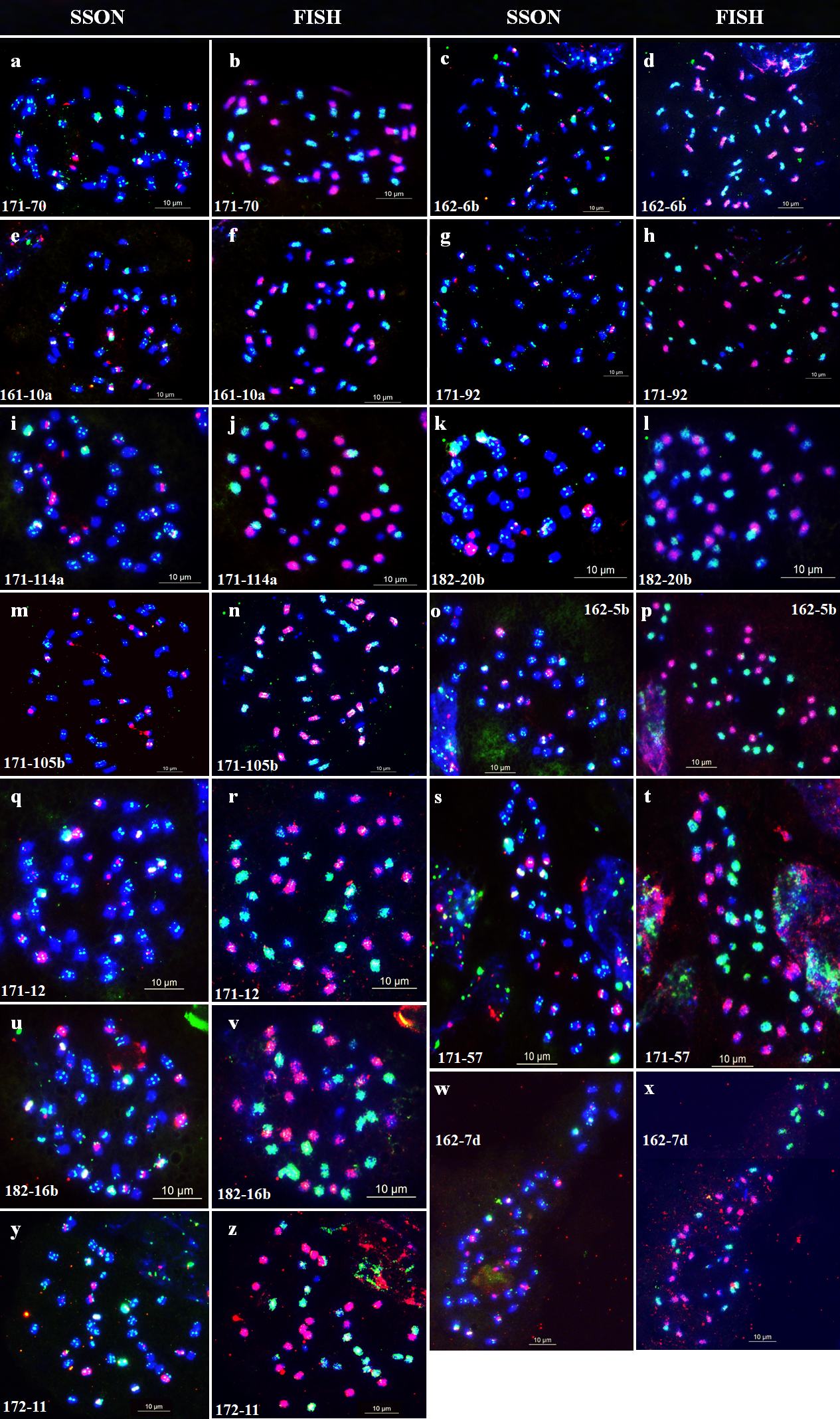

Supplement: Supplementary file 5 — Additional file 5: Fig. S5. Chromosome aberration variants detected in the radiation-induced M1 plant 161-1a of peanut cultivar Silihong (SLH) after sequential FISH/GISH. FISH using Multiplex #3 (a, c, e, g, i, k, m, o, q, s, u, w and y); GISH using A. duranensis genomic DNA (green) and A. ipaensis genomic DNA (red) (b, d, f, h, j, h, j, l, n, p, r, t, v, x and z). Scale bar: 10 μm. [file 12870_2021_2875_MOESM5_ESM.tif]
